# Controlling invasive Argentine ants, *Linepithema humile*, in conservation areas using horizontal insecticide transfer

**DOI:** 10.1038/s41598-019-56189-1

**Published:** 2019-12-20

**Authors:** Grzegorz Buczkowski, Theresa C. Wossler

**Affiliations:** 1 Department of Entomology, Purdue University, West Lafayette, IN 47907 USA; 2 Department of Botany and Zoology, Stellenbosch University, Private Bag X1, Matieland, 7602 South Africa

**Keywords:** Conservation biology, Invasive species

## Abstract

Invasive ants are major agricultural and urban pests and a significant concern in conservation areas. Despite long history of control and eradication efforts, invasive ants continue to spread around the globe driven by a multitude of synergistic factors. Lack of effective management tools is one of the biggest challenges in controlling invasive ants. The goal of the current study was to improve the efficacy and safety of ant management and to develop effective control strategies for sensitive conservation areas. We utilized the Argentine ant (*Linepithema humile*) as a model system to evaluate a target-specific pesticide delivery system that exploits the interconnected nature of social insect colonies to distribute a toxicant effectively within the colony. The approach, based entirely on horizontal transfer, takes advantage of various levels of social interactions in ant colonies to disseminate a toxicant throughout the colony. Results of laboratory studies coupled with LC/MS/MS analysis demonstrate that fipronil is toxic to Argentine ants in extremely small (nanogram) quantities and is efficiently transferred from a single treated donor to multiple recipients, causing significant secondary mortality. A field study was conducted in native fynbos plots invaded by Argentine ants. The study consisted of collecting naïve workers, treating them with fipronil, and releasing them within invaded plots. Results show that the release of fipronil-treated ants reduced Argentine ant abundance by >90% within 24 h. The horizontal transfer approach offers environmental benefits with regard to pesticide use in ecologically sensitive environments and appears ideally suited for ant management in conservation areas.

## Introduction

Invasive ants continue to spread globally driven by multiple factors including globalization^1,2^, climate change^3^, secondary invasions^4,5^, lack of effective management tools^6^, poor understanding of biology and natural history^7^, and a variety of technical and political factors^8^. Among invasive ants, the Argentine ant, *Linepithema humile*, is one of the most widespread and damaging invaders^9,10^. This is due to its global distribution, high local abundance, and high potential to cause ecological and economic damage^9,11,12^. Argentine ants are mainly associated with anthropogenic environments and are frequently a pest in urban environments^13^. However, Argentine ants also invade native habitats with relatively little human disturbance^14–16^. In South Africa, Argentine ants have invaded the Cape Floral Region, one of the world’s most iconic centers of plant biodiversity^17,18^.

The effective management of invasive ants is constrained by a number of factors, many relating to their social and spatial structure^8^. A major issue is that many protocols for ant management are not truly based on ant biology^19^ and the prevailing treatment strategies and product labels are not entirely compatible with the ant’s biology and unicolonial population structure^8^. Many species have high-density polygynous populations and colonies often nest in inaccessible places that are protected from direct pesticide applications. Furthermore, many ant species are highly polydomous, comprised of multiple, spatially dispersed nests further complicating efforts to find and effectively treat all nests. Locating nests prior to treatment is not practical in most situations, especially natural environments where terrain and ground cover limit access. Instead, the insecticide is typically applied in areas where ants are expected to nest and/or forage^19,20^. Foraging workers subsequently visit the treated areas and translocate the insecticide to other members of the colony, especially individuals that do not forage (queens) or cannot forage (larvae) independently. However, most spray insecticide treatments deployed for ant control result in only a small proportion of foraging workers being killed directly and control is often incomplete and resurgences are common^21^.

While the high-density and interconnected nature of Argentine ant supercolonies is a major impediment to their effective control, it may also offer opportunities for improving management approaches. The cohesion, efficiency, and survival of ant colonies is dependent on interaction networks and exchanges which require direct contract among individuals^22^. In situations where individuals are exposed to pesticides, direct contact among individuals may promote insecticide spread through horizontal transfer. Horizontal transfer occurs when foraging individuals acquire the insecticide at the point of application and subsequently transfer it to other members of the population^23^ through various social behaviors, most notably allogrooming. Subsequently, horizontal transfer may result in secondary mortality if a lethal dose is transferred from exposed donors to unexposed recipients.

To improve the efficacy and safety of ant management more research is needed on new treatment products, delivery methods, and technologies. One of the most important goals is to develop effective ant control strategies for sensitive conservation areas. The main goal of such efforts is to minimize negative environmental impact, specifically pesticide residues that might have non-target effects. Recent developments in this area include hydrogel baits^24–26^, prey-baiting based on the use of insecticide-treated prey^27,28^, and pheromone-assisted baiting^29,30^. Another new development is exploiting social interactions within colonies to promote the spread of insecticides through horizontal transfer. A recent study evaluated a novel control method based on horizontal transfer for controlling black carpenter ants, *Camponotus pennsylvanicus*^31^. A three-step method consisting of trap-treat-release was used in field experiments and demonstrated that fipronil is effectively transferred when foraging workers are trapped, sprayed with insecticide, and released back into their colonies. Fipronil was effectively vectored from treated donor ants to untreated recipients and caused significant secondary mortality.

The goal of the current study was to build on work described in^31^ and evaluate a target-specific pesticide delivery system that relies on horizontal transfer to control Argentine ants in South African fynbos. The first objective was to perform laboratory studies to generate quantitative information on factors affecting horizontal transfer, specifically the amount of fipronil transferred from treated to untreated ants and the number of untreated ants killed in interactions with a single treated ant. The second objective was to utilize information obtained in laboratory experiments to examine horizontal transfer in areas invaded by Argentine ants and to determine the feasibility of the horizontal transfer approach for practical pest control.

## Methods

### Horizontal transfer of fipronil – analytical study using LC/MS/MS

Colonies of Argentine ants, *Linepithema humile*, were collected in Winston-Salem, North Carolina and transported to the laboratory at Purdue University. Colonies were reared on 25% sucrose solution provided *ad libitum* and freshly killed cockroaches twice a week. Colonies were maintained and all experiments were conducted at 27 ± 2 °C, 60 ± 10% RH, and 14:10 L:D cycle. Experimental colonies were set up by aspirating 100 workers from stock colonies and transferring them to 25 × 30 × 9 cm high Fluon-coated plastic boxes. The ants were allowed 48 h to acclimate to an artificial nest consisting of a plastic Petri dish (5 cm diameter) filled with moist dental plaster. Food consisting of 15% sucrose water was provided during the acclimation period and during the test. At the end of the acclimation period, a single worker (donor) obtained from conspecific colonies was introduced into the recipient colony. To differentiate donors from recipients, the donors were marked with a small dot of acrylic paint (Testors Craft, Rust-Oleum Corp, Vernon Hills, IL) on the abdomen. The donors were sprayed with Termidor SC (9.1% fipronil, BASF Corporation, Research Triangle Park, NC). The spray solution was prepared by mixing 6.3 mL of Termidor with 1 liter of water resulting in 0.06% fipronil solution. The 0.06% concentration is the label rate recommended for controlling pest ants. To prepare the donors, 20 worker ants were placed inside a plastic Petri dish (9 cm diameter). The inner side of the dish was coated with Fluon to prevent escapes. The spray solution was delivered using an atomizer (Specialty Bottle, Seattle, WA). A single pump from the atomizer, equivalent 150 µL of 0.06% Termidor, was delivered to the dish so that all ants were uniformly coated with a thin layer of spray solution. To estimate the amount of fipronil present on the donors, 12 randomly selected ants were removed from the treatment dish, placed in individual glass tubes, and kept in −20 °C freezer until analysis. The remaining ants were transferred to nest boxes containing 100 recipient workers, one donor per recipient colony (n = 6). To estimate the amount of fipronil transferred from donors to recipients, 10 dead recipient ants were randomly selected from each of six replicates, 24 h after introducing the donors. For LC/MS/MS analysis, each recipient ant was placed into a 1.7 mL centrifuge tube and 0.1 mL of acetonitrile extraction solvent containing 300 ng/mL of ^13^C_4_-fipronil (Sigma Aldrich, St. Louis, MO) was added to each ant. The ants were ground using plastic pestles, vortexed, and sonicated for 10 minutes, then centrifuged at 10,000 rpm × 5 minutes. The supernatant was collected and stored at −20 °C. The extracts were analyzed using LC/MS/MS at the Metabolite Profiling Facility at Bindley Bioscience Center at Purdue University. An Agilent 1200 Rapid Resolution liquid chromatography (LC) system coupled to an Agilent 6460 series QQQ mass spectrometer (MS) was used to analyze fipronil in each sample. An Agilent Zorbax SB-Phenyl 2.1 mm × 100 mm, 3.5 µm column was used for LC separation (Agilent Technologies, Santa Clara, CA). The buffers were: (a) water +0.1% formic acid and (b) acetonitrile +0.1% formic acid. The linear LC gradient was as follows: time 0 minutes, 10% B; time 0.5 minutes, 10% B; time 8 minutes, 100% B; time 10 minutes, 100% B; time 11 minutes, 10% B; time 15 minutes, 10% B. The flow rate was 0.3 mL/min. Multiple reaction monitoring was used for MS analysis. The jet stream ESI interface had a gas temperature of 325 °C, gas flow rate of 7 L/minute, nebulizer pressure of 45 psi, sheath gas temperature of 250 °C, sheath gas flow rate of 7 L/minute, capillary voltage of 3500 V in negative mode, and nozzle voltage of 500 V. The ΔEMV voltage was 400. All data were analyzed with Agilent Masshunter Quantitative Analysis (Version B.06.00). A linear calibration curve was prepared from 0.2–1500 ng/mL for fipronil and ^13^C_4_-fipronil was used as internal standard and for absolute quantitation. The retention time of fipronil was 8.97 minutes. The 434.7 → 329.9 (fipronil)/438.7 → 333.9 m/z (^13^C_4_-fipronil) transition was used for quantitation purposes.

### Horizontal transfer of fipronil – laboratory study

The goal of the laboratory study was to determine the number of Argentine ants killed in interactions with a single treated donor ant. This was accomplished by placing a single treated donor in colonies of progressively larger size. Experimental colonies were set up by aspirating workers from stock colonies and transferring them to 25 × 30 × 9 cm high Fluon-coated plastic boxes. Three colony sizes were tested: 100, 250, and 500 workers and six replicates were performed for each colony size. The ants were allowed 48 h to acclimate to an artificial nest consisting of a glass test tube (25 mm diameter × 150 mm long) half filled with water. The test tube was stoppered with a cork that contained a single hole to allow entry and wrapped in aluminum foil to keep it dark. Food consisting of 25% sucrose solution was provided during the acclimation period and during the test. At the end of the acclimation period, a single donor worker obtained from a conspecific colony was introduced into the recipient colony. To differentiate donors from recipients, the donors were marked with a small dot of acrylic paint on the abdomen as above. Donor ants were then treated with fipronil (0.06% Termidor SC) using direct spray as above and immediately transferred to nest boxes containing the recipients. Control tests (n = 6) consisted of colonies of 100, 250 or 500 workers provided with a single ant sprayed with water alone. Mortality in the donor and the recipients was determined at 2, 4, 8, 16, 24, 48, 72, and 96 hours.

### Targeted fipronil delivery – field study

Field plots containing colonies of *L. humile* were established at Helderberg Nature Reserve, Somerset West, Western Cape, South Africa (−34.06S, 018.87E). The plots were 10 by 10 m and were separated by at least 25 m buffer zones. To estimate initial ant densities (day 0) the plots were sampled using note cards baited with a blend of canned tuna and corn syrup^32^. Within each plot, the bait cards were placed along two transects, 10 m long perpendicular lines forming a cross through the center of each plot. Eight evenly spaced cards were used along each transect (16 baits per plot). The cards were placed on the ground and collected 1 hr after placement to estimate the number of Argentine ants present. Following census baiting, each 100 m^2^ plot was provisioned with 10,000 treated donor ants (equivalent to 5.0 grams of workers) which had been topically sprayed with 0.06% fipronil. The number of donor ants selected was based on results obtained in LC/MS/MS analysis and laboratory transfer tests. Donor ants were collected within Helderberg Nature Reserve in areas outside of experimental plots, extracted from nesting material, placed in a plastic box, and sprayed using an atomizer. Each pump from the atomizer delivers 150 µL of spray solution (±5%). Ten pumps from the atomizer were delivered for each box so all the ants were uniformly coated with a thin layer of the spray solution. Therefore, a total of 1.5 mL of fipronil solution was applied to each box. This is equivalent to 1 gallon solution per 1,000 square feet (40 mL per sq m), the recommended application rate for Termidor. Following treatment, the ants were immediately released into the center of the experimental plot by gently emptying the container onto the ground and allowing them to naturally disperse. Efficacy of fipronil transfer was examined on days 1, 3, 7, 14, and 21 using baited note cards as above. Six experimental plots and four control plots were established. All assessments were performed from February to March 2019.

### Statistical analysis

The effect of colony size and time since exposure to fipronil treated nestmates on worker mortality in the laboratory was assessed using an ANOVA test. A two-way Repeated Measures ANOVA was used to assess the change in worker abundance over time under field conditions. The Greenhouse-Geisser correction was applied due to violations of the assumption of sphericity (Mauchly’s test) and equality of variances (Levene’s test). This test recalculates new degrees of freedom in order to obtain a valid *F-ratio*. Tukey’s HSD test was used for posthoc pairwise comparisons and parameter estimates showed changes in worker abundances relative to the reference group. Statistical significance was set at α = 0.05 and all analyses were performed using Statistica 13.2^33^.

## Results

### Horizontal transfer of fipronil – analytical study using LC/MS/MS

**Figure 1:**
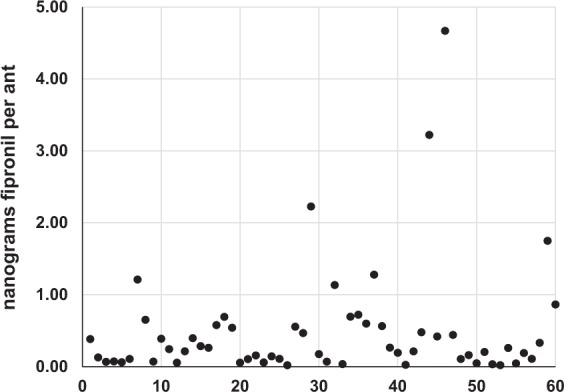
Nanograms of fipronil detected in recipient ants in tests where 100 untreated recipients interacted with a single treated donor. Each dot represents a single ant. Results for all 60 recipients are presented (6 replicates of 10 randomly selected recipients) in sets of 10 from left to right.

Results of the laboratory study on the horizontal transfer of fipronil coupled with LC/MS/MS analysis revealed that fipronil was efficiently transferred from treated donors to untreated recipients. The mean amount of fipronil detected on the donors was 78 ± 17 ng (range 39–94 ng; Fig. 1). The mean amount of fipronil detected on the recipients was 0.49 ± 0.79 ng (range 0.02–4.67 ng; Fig. 1). As expected, the mean amount of fipronil present on the donors was significantly higher than the amount present on the recipients (t-test, *t* = 34.9, *df* = 70, *P* < 0.001). The amount of fipronil detected on the recipients was highly consistent and less than one nanogram was detected on 53 out of 60 (88%) recipient ants (Fig. 1). The other recipients had fipronil levels ranging from 1 to less than 5 ng. Results demonstrate that fipronil applied via spray applications was readily transferred from treated to untreated workers and that fipronil is toxic to Argentine ants in extremely small amounts.

### Horizontal transfer of fipronil – laboratory study

Laboratory experiments demonstrated that fipronil is relatively fast-acting and readily transferred from a single donor to multiple recipients (Table 1). All donors died within 2 h of treatments. Mortality in the recipient ants was significantly influenced by colony size (ANOVA, *F* = 106.3, *df* = 2, *P* < 0.001), time (ANOVA, *F* = 1002.5, *df* = 5, *P* < 0.001), and treatment (ANOVA, *F* = 2923.6, *df* = 1, *P* < 0.001), and a significant three-way interaction was detected among all factors (Wilk’s test, *F*_(*10, 40*)_ = 13.2, *P* < 0.001). Mortality increased greatly between 16 and 24 h in tests with all colony sizes, and the majority of ants died within the first 24 h. In tests with 100 recipients, 100% mortality was achieved in 24 h and in tests with 250 recipients 100% mortality was achieved in 72 h. These results demonstrate that a single donor is capable of transferring a lethal dose of fipronil to at least 250 nestmates. In tests with 500 recipients, mortality reached 61 ± 3% in 96 h. Because mortality did not reach 100% and largely leveled off after the first 24 h it was possible to determine the maximum number of workers that can be killed in interactions with a single donor. Results show that a single donor ant is capable of killing 307 ± 15 nestmates (range 260 to 359 ants). Mortality in control tests did not exceed 6%.

**Table 1:** Mean cumulative percent mortality (±st dev) in *L. humile* workers exposed to a single fipronil-treated nestmate.

Treatment	Colony size	Time (hours)							
		2	4	8	16	24	48	72	96
control	100 workers	0 ± 0 a, a	0 ± 0 a, a	0 ± 0 a, a	1 ± 1 a, a	3 ± 1 a, a	5 ± 1 a, a	6 ± 2 a, a	6 ± 2 a, a
control	250 workers	0 ± 0 a, a	0 ± 0 a, a	0 ± 0 a, a	0 ± 0 a, a	1 ± 0 a, a	1 ± 1 a, a	2 ± 1 a, a	2 ± 1 a, a
control	500 workers	0 ± 0 a, a	0 ± 0 a, a	0 ± 0 a, a	0 ± 0 a, a	1 ± 0 a, a	2 ± 0 a, a	3 ± 0 a, a	3 ± 0 a, a
fipronil	100 workers	0 ± 0 a, a	0 ± 0 a, a	8 ± 1 a, a	27 ±± 5 b, a	100 ± 0 b, a	100 ± 0 b, a	100 ± 0 b, a	100 ± 0 b, a
fipronil	250 workers	0 ± 0 a, a	0 ± 0 a, a	9 ± 3 ab, a	19 ± 5 b, b	86 ± 6 b, b	98 ± 3 b, a	100 ± 0 b, a	100 ± 0 b, a
fipronil	500 workers	0 ± 0 a, a	0 ± 0 a, a	5 ± 2 a, a	9 ± 1 ab, c	42 ± 3 b, c	55 ± 4 b, b	59 ± 3 b, b	61 ± 3 b, b

Means followed by the same letter are not significantly different (*P* ≤ 0.05) by Tukey’s HSD test. First letter indicates comparisons across treatments (control vs. fipronil), second across colony sizes within a treatment.

### Targeted fipronil delivery – field study

**Figure 2:**
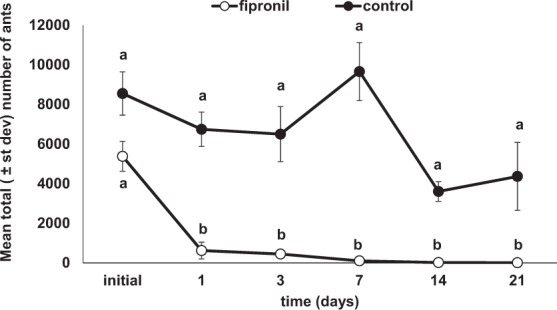
Mean total (±st dev) number of *L. humile* workers detected within field plots provisioned with fipronil-treated or water-treated (control) nestmates. Letters indicate pairwise differences in ant abundance at each assessment time between fipronil-treated and control plots.

The release of fipronil-treated ants significantly reduced *L. humile* abundance over time relative to untreated control plots (Fig. 2). The number of *L. humile* detected over time differed significantly between treated and control plots (Wilk’s test, time × treatment: *F*_(*5, 4*)_ = 167.5, *P* < 0.001). At the start of the treatment, no difference in ant abundance was found between control and fipronil-treated plots (ANOVA, *F*_(*1, 8*)_ = 118.6, *P* = 0.06, Fig. 2), but a significant decline in the abundance of ants in the fipronil-treated plots was observed within 1 day of treatment (ANOVA, *F* = 1048.9, *P* < 0.0001, Fig. 2). The number of ants in the treated plots differed significantly from controls at each assessment time after treatment (Fig. 2). The decline in ant abundance in the treated plots was relatively fast and the abundance of *L. humile* declined by 91 ± 14% within 1 day of releasing the treated ants. Ant abundance in the control plots varied throughout the study depending on weather conditions and changing foraging patterns.

## Discussion

The current study evaluated a novel approach for controlling invasive ants, based solely on horizontal transfer. The approach takes advantage of various levels of social interactions in ant colonies to effectively distribute a toxicant within the colony and ultimately cause colony demise.

Fipronil is toxic to Argentine ants in extremely small amounts, is efficiently transferred from treated donor ants to untreated recipients, and causes significant secondary mortality, as demonstrated in the laboratory study coupled with LC/MS/MS analysis. In colonies consisting of 100 workers and provisioned with a single treated donor, 100% mortality was achieved in 24 h. LC/MS/MS analysis revealed that donor ants had an average of 78 ng of fipronil and recipient ants had an average of 0.5 ng. This suggests that a single donor treated via direct spray application is capable of transferring a lethal dose to at least 156 ants, highlighting fipronil’s toxicity and potential for transfer. The actual amount of fipronil required to kill a single Argentine ant worker may actually be much lower than 0.5 ng. Laboratory tests involving colonies consisting of 500 recipients demonstrated that a single donor ant is capable of transferring a lethal dose of fipronil to an average of 307 recipients. Therefore, the amount of fipronil required to kill a single Argentine ant may actually be closer to 0.25 ng. These results agree with Rust *et al*.^21^ who estimated that the LD_50_ for topically applied fipronil is 0.39 ng per Argentine ant worker. The majority of fipronil applied to the donors was passed to untreated nestmates through horizontal transfer. Various behavioral mechanisms may have played a role in horizontal transfer depending on whether the donor ants were alive or dead. Direct contact, mutual grooming, and oral transfer of fipronil that may have been accidentally ingested by the donor while self-grooming and subsequently shared with the recipients by trophallaxis likely facilitated the transfer of fipronil from live donors to the recipients. Physical contact and necrophoresis (carrying of dead nestmates) likely played a role in interactions involving dead donors. High necrophoretic activity has been shown to play an important role in the horizontal transfer of fipronil^23^. The horizontal transfer of fipronil may have continued beyond secondary kill and involved higher levels such as tertiary mortality whereby recipients become secondary donors and vector the insecticide to previously unexposed nestmates. Tertiary mortality played a significant role in the horizontal transfer of fipronil in carpenter ants^31^. In a laboratory test, 50 new workers were exposed to 20 workers that died from having contact with a single primary donor and 90% of the workers died in 96 h^31^. Because fipronil is toxic in ultra-low (ng) amounts, non-repellent, and readily transferrable among ants, it is likely that tertiary mortality played an important role in the current study. Presumably, workers encounter dead nestmates, take them to the refuse pile, obtain a lethal dose while carrying the dead nestmate, return to the nest and die, and are subsequently carried to the refuse pile by another member of the colony for the process to repeat until workers engaging in necrophoresis receive sublethal doses of fipronil.

In the field study, the release of fipronil-treated ants reduced Argentine ant abundance by >90% within 24 h. The decline was relatively fast, with the rate and the level of mortality similar to laboratory studies. This suggests that similar behaviors and processes, including secondary and tertiary transfer, may play a role in the field. Laboratory tests demonstrated that a single treated worker is capable of killing approximately 307 nestmates. This suggests that the release of 10,000 treated ants could potentially affect 3,070,000 ants. However, relative to laboratory studies, horizontal transfer in the field is likely less efficient due to more complex environment, variable weather conditions, and other factors. The exact number of ants present within the plots was impossible to determine because the nests were highly dispersed, inaccessible (below ground and/or within logs), and frequently moved. Additionally, the number of ants within the plots may have fluctuated considerably given that resident ants could freely leave the plots and non-resident ants could freely enter the plots from adjacent areas. The exact fate of the donor ants was unknown as well as they quickly disappeared into the substrate after being released. It’s unclear whether they dispersed over the whole plot or remained closer to the central release point, how many died on the surface vs. below ground, and how many actually interacted with resident ants vs. remained undiscovered. Laboratory observations showed that treated workers stay alive for approximately 2 h before becoming symptomatic and unable to walk. The walking speed of Argentine ant workers is approximately 2 cm per sec^34^, equivalent to 108 meters per hour. Given that treated workers stay alive for at least 2 hours, they could have easily dispersed throughout the plots. In fact, some of the treated ants may have left the experimental plots and affected nests in nearby untreated areas. This may have helped achieve satisfactory control in the experimental plots as influx of untreated workers into treated plots is typically a problem in field studies where relatively small treated areas are surrounded by vast untreated areas.

Fipronil is highly toxic to Argentine ants, relatively fast-acting, and effective in extremely low quantities. Assuming that 0.25 ng fipronil is sufficient to kill a single Argentine ant, a bottle of Termidor SC (2.3 liter size, formulated at 9.1% fipronil by weight, equivalent to 221 grams of fipronil per bottle) is capable of killing approximately 900 billion Argentine ants, equivalent to 47 metric tons of ants assuming that a single ant weighs 0.52 mg. It is estimated that the Large Supercolony of Argentine ants spanning 1,000 km from San Francisco to the Mexican border in California contains approximately one trillion individuals^35^. According to the label, a single bottle is enough to prepare 380 liters of 0.06% solution to be applied over 6,200 square meters, or a 78 × 78 m plot, highlighting the inefficiency of current management approaches. It is estimated that less than 0.1% of pesticides applied for pest control reach their target^36^. The rest remain in the environment often resulting in environmental pollution and non-target effects. The transfer approach evaluated in the current study precisely delivers the insecticide to the target species, greatly reduces pesticide use while increasing target specificity, and completely eliminates the need for direct insecticide applications to the environment. Therefore, it might be particularly useful for controlling invasive ants in sensitive areas such as national and state parks, nature reserves and wilderness areas where invasive ants must be carefully managed to avoid non-target effects.

In summary, the current study evaluated a novel, target-specific approach for controlling invasive ants based on the principles of horizontal transfer. Future studies should aim to further optimize the transfer approach. One option might be to use treated brood instead of treated workers to vector toxicant throughout the population. Brood is extremely important for ant colonies and modulates a number of processes on both the individual and the colony level^37^. Ants are highly protective of their brood and exposed brood is immediately moved to the safety of the nest. Releasing treated brood might assure that the toxicant is placed where it’s likely going to be the most effective – below ground and in close proximity to the queens which might help target the reproductive caste and prevent resurgences. Another option might be to release treated workers from a mutually-aggressive supercolony to ensure more ant-to-ant contact. In the current study, horizontal transfer was achieved through peaceful behaviors such as mutual grooming and trophallaxis. However, the efficiency of transfer might increase if the toxicant is vectored via aggressive, rather than peaceful interactions. Argentine ants are highly aggressive towards non-nestmates from distinct supercolonies^38,39^ and multiple workers typically cooperate in fighting a single non-nestmate which might accelerate the spread of the toxicant.
